# Processing Techniques for Bioresorbable Nanoparticles in Fabricating Flexible Conductive Interconnects

**DOI:** 10.3390/ma11071102

**Published:** 2018-06-28

**Authors:** Jiameng Li, Shiyu Luo, Jiaxuan Liu, Hang Xu, Xian Huang

**Affiliations:** Department of Biomedical Engineering, Tianjin University, 92 Weijin Road, Tianjin 300072, China; MrLeepursuit@163.com (J.L.); luoshiyu@tju.edu.cn (S.L.); jiaxuanliu@tju.edu.cn (J.L.)

**Keywords:** bioresorbable electronics, printing electronics techniques, conductive inks

## Abstract

Bioresorbable electronics (or transient electronics) devices can be potentially used to replace build-to-last devices in consumer electronics, implantable devices, and data security, leading to reduced electronic waste and surgical processes through controllable dissolution. Recent development of printing bioresorbable electronics leads to bioresorbable conductive pastes or inks that can be used to make interconnects, circuit traces, and sensors, offering alternative solutions for the predominant complementary metal oxide semiconductor (CMOS) processes in fabrication of bioresorbable electronics. However, the conductivities offered by current bioresorbable pastes and processing techniques are still much lower than those of the bulk metals, demanding further improvement in both paste composition and process optimization. This paper aims at exploring several influential factors such as paste compositions and processing techniques in determining conductivities of bioresorbable patterns. Experimental results reveal that an optimized paste constituent with a ratio of Zn:PVP:glycerol:methanol = 7:0.007:2:1 by weight can generate stable conductive pastes suitable for a screen printing process. In addition, a high conductivity of 60,213.6 S/m can be obtained by combining hot rolling and photonic sintering. The results demonstrate that large-scale transient electronics can be obtained by combining screen printing, hot rolling and photonic sintering approaches with optimized paste compositions, offering important experimental proofs and approaches for further improving the conductivity of bioresorbable pastes or inks that can accommodate the demands for mass fabrication and practical use in electronic industry.

## 1. Introduction

The majority of more than 50 million tons of electronic-waste generated each year globally end up in the landfill, or are just simply incinerated, causing enormous environmental issues, such as soil compaction, acid rain, and water pollution [1,2]. Efforts in recycling electronic-waste have been focused on reducing the cost and time consumption of recycling processes, which features a few iconic techniques such as automatic sorting, mechanical disassembly, and magnetic separation [3,4,5]. However, these techniques have stringent requirements for large facility, expensive equipment, and hazardous chemicals. Recent development of transient electronic devices that can degrade under environmentally friendly approaches triggered by water, humidity, light, and air flow leads to a safe and effective solution to the rampant pollution caused by electronic-waste while facilitating recycling [6,7,8,9,10,11].

Bioresorbable electronic devices have been presented in various formats with applications ranging from data storage to internal medicine and health care [12,13,14,15,16,17]. The fabrication approaches of such devices involve modified complementary metal oxide semiconductor (CMOS) processes and printing electronics techniques [18]. An emerging trend combines CMOS and printing electronics technology to yield bioresorbable circuits that offer both improved complexity and high time/cost efficiency, demonstrating promising use in replacing build-to-last electronic devices for specific applications that only require short working periods [19]. Fabrication of bioresorbable electronic devices can be achieved by anhydrous and low temperature processes at the cost of device performance [20]. Thus, improvement of CMOS and printing electronics technology for bioresorbable electronics is critically needed.

One fundamental improvement involves developing bioresorbable pastes or inks for making interconnectors, circuit traces, and other electronic components such as sensors, resistors, and electrodes [12,13,14,15,16,17]. Using zinc nanoparticles (Zn NPs) together with various printing and sintering approaches, bioresorbable conductive patterns has been demonstrated in our previous studies as well as other literature [19,20,21,22,23]. However, existing bioresorbable inks offer conductivity ranging from 2.2 × 10^4^ to 3.0 × 10^5^ S/m [19,20,21,22], which is at least 20 times lower than that of bulk metal. Improved conductivity may be obtained by adjusting compositions of bioresorbable inks as well as other handling techniques. In this paper, we investigate the influence of weight ratio of Zn NPs as well as a new process flow that involves combination of screen printing, hot rolling and photonic sintering techniques. The influence of individual technique in determining the conductivities of Zn patterns was also investigated. A high conductivity at 60,213.6 S/m can be achieved with excellent flexibility to withstand repeated bending. The results suggest that large-scale transient electronics can be obtained by combining screen printing, hot rolling and photonic sintering approaches with optimized ink compositions, offering important experimental proof and approaches for further improving the conductivity of bioresorbable pastes and inks that can adapt to the demands for mass fabrication and practical use in electronic industry.

## 2. Materials and Methods 

### 2.1. Preparation of Conductive Inks and Bioresorbable Substrates

Preparation of Zn nanoparticle inks started with mixing glycerol and methanol at a mass ratio of 2:1 to yield a bi-solvent system. Polyvinylpyrrolidone (PVP, 0.1 wt% for Zn NPs) was then dissolved in the mixed solvent as surfactant to increase ink viscosity and prevent aggregation of Zn NPs. Zn NPs (50 nm in diameter, Beijing Dk Nano technology Co., Ltd., Beijing, China) were then added to the solution at a 7:3 weight ratio, followed by mechanical stirring and sonication for 30 min, resulting in a conductive paste with a proper viscosity (~10 Pa·s) and particle sizes (~500 nm) to satisfy the requirements of screen printing. 

Polyvinyl alcohol (PVA) has been used for making bioresorbable substrates. A solution of PVA can be obtained by adding 10 wt% PVA (Energy Chemical Inc., Shanghai, China) to DI water at 80 °C and stirring at 500 rpm until the solution became bubble free. The resulting solution was subjected to filtration (membrane size is 500 nm) to remove any insoluble impurity, and was then dispersed onto glass slides, allowing the formation of flexible and transparent films after drying in air at room temperature for 24 h. 

### 2.2. Fabrication of Conductive Patterns

**Figure 1:**
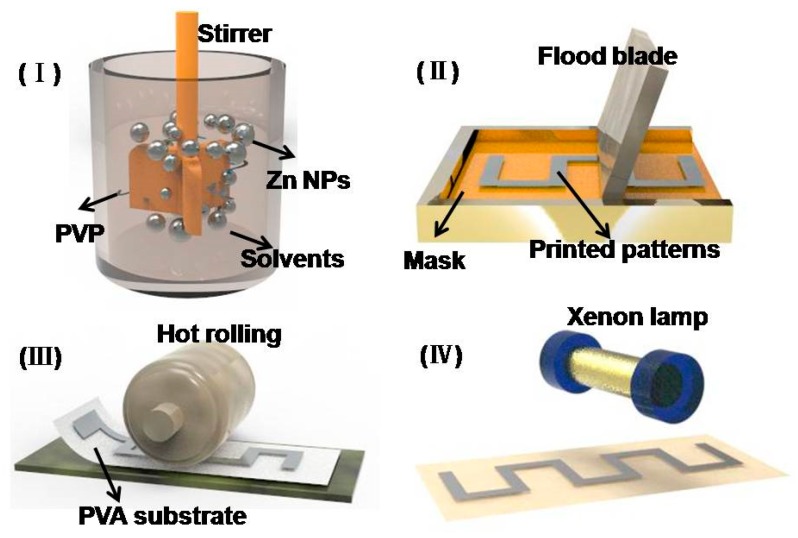
Schematics of fabricating transient electronic patterns through ink preparation (**I**), screen printing (**II**), hot rolling; (**III**) and photonic sintering; (**IV**) approaches.

Figure 1 demonstrates a fabrication process for printing bioresorbable patterns made of Zn NPs on a PVA substrate. The entire process involves screen printing, hot rolling and photonic sintering, all of which are anhydrous to prevent Zn NPs from reacting with water. Curing and sintering of as-printed patterns were conducted under the protection of argon to avoid surface oxidation of Zn NPs. A screen printer (PHP-2020A, Shanghai Xuanting Co., Ltd., Shanghai, China) was used to print various patterns ranging from straight interconnect to curved electrodes, followed by a curing process at 100 °C to remove solvent in the patterns. A hot roller (MRX-JS200L, Shenahen Mingruixiang Automation Equipment Co., Ltd., Shenzhen, Guangdong, China) with a rolling speed of 40 mm/s was used to compress the printed patterns to remove additional void space generated because of solvent evaporation. The patterns were then sintered by a photonic sintering system (LH840, Xenon Co., Ltd., Dover, DE, USA) using an energy at 4.9 J/cm^2^ per pulse. The morphology of the conductive patterns was measured by a scanning electron microscopy (SEM, SUPRA55VP, Zeiss, Oberkochen, Baden-Württemberg, Germany) with a 15 KV accelerating voltage and a working distance of 10 mm, and the conductivity of patterns was measured through a four-point probe measurement system (FPPM2015A, Suzhou Jingge Electronic Co., Ltd., Suzhou, Jiangsu, China) following the ASTM F1711-96(2008) standard.

## 3. Results and Discussion

**Figure 2:**
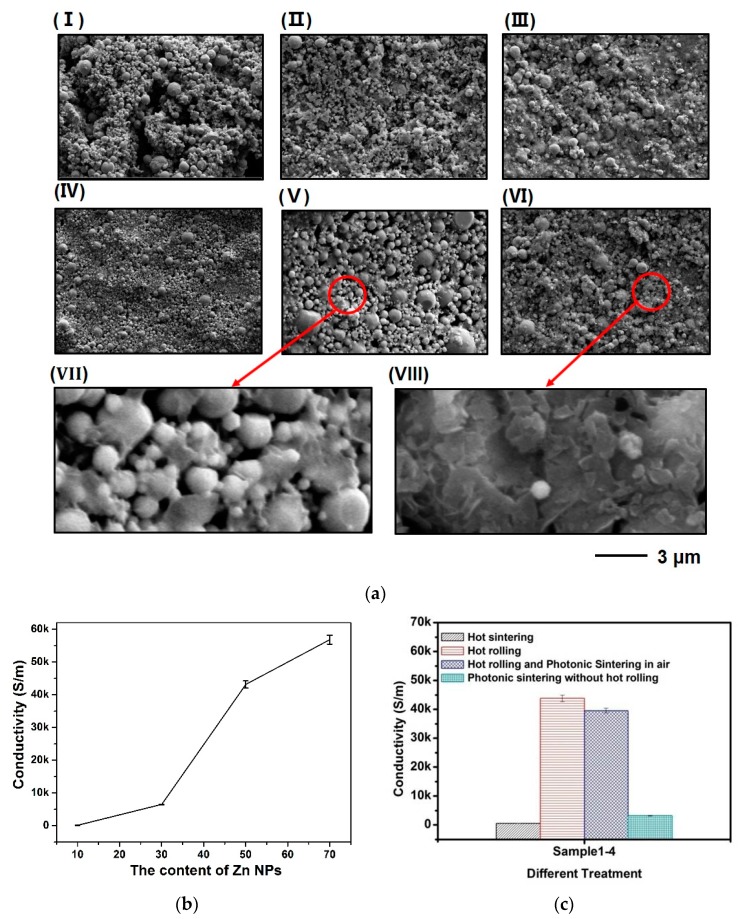
(**a**) The surface morphology of printed patterns using different content of Zn NPs at (**I**) 10 wt%, (**II**) 30 wt%, (**III**) 50 wt%, and (**IV**) 70 wt%. (**V**) and (**VI**) represent that patterns treated without and with hot rolling, respectively. (both of them without photonic sintering). (**VIII**) and (**VII**) represent amplified images of (**VI**) and (**V**), respectively; (**b**) The conductivity of patterns using different content of Zn NPs; (**c**) The conductivity of printed pattern under different treatments.

An optimized weight ratio between Zn NPs and the mixed solution was first investigated. The weight ratio of Zn NPs is considered as a determining factor that significantly influences the conductivity of bioresorbable patterns. The conductive patterns obtained by pastes with varied weight ratios of Zn NPs from 10% to 70% were fabricated by screen printing and were treated with hot-rolling and photonic sintering. The conductivities of sintered patterns improve with increased ratios of Zn NPs (Figure 2b). Patterns made by the conductive paste with 10 wt% Zn NPs are only 132.8 S/m, while patterns with 50 wt% and 70 wt% Zn NPs show good conductivities of 43,144.2 and 56,849.5 S/m, respectively. SEM images of the above patterns further show increased density and reduced porosity in bioresorbable patterns with higher weight ratios (Figure 2(aI–aIV)). The sample made by the conductive paste with 70 wt% Zn NPs shows the highest conductivity, and, thus, this weight ratio is used throughout our following investigation.

It is also observed that subsequent treatments of the printed patterns after screen printing perform important roles in determining the conductivity of the patterns. Conductivity of printed patterns that have been subjected to pure hot rolling is determined to be 42,806.9 S/m as compared with 594.9 S/m for an as-printed sample. The result suggests that increasing the compactness of printed samples by hot rolling is an effective approach to achieve high conductivity. When no further sintering processes is involved, the major improvement of the conductivity can be attributed to the volume shrinkage that can increase spontaneous bonding of nanoparticles and enhance tunneling effect among nanoparticles. SEM images of patterns with (Figure 2(aVI–aIII)) and without (Figure 2(aV–aVII)) hot rolling further demonstrate that significant coalescence of nanoparticles exists in the hot-rolled sample.

**Figure 3:**
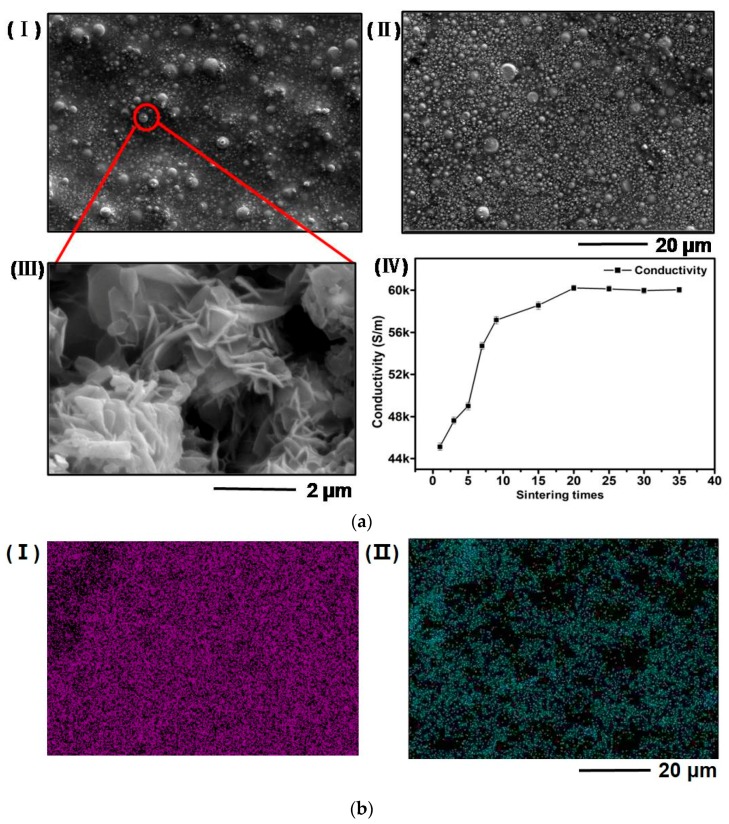
(**a**) (**I**) and (**II**) represent that patterns treated with 20 and 25 light pulses, respectively; (**III**) a higher resolution of SEM image of surface morphology of Zn NPs after treating with 20 pulses of photonic sintering; (**IV**) The changes of conductivity with different number of light pulses in the photonic sintering process; (**b**) The mapping of a printed pattern shows the distribution of Zn (**I**) and O (**II**).

The effect of sintering approaches was also investigated by comparing prolonged thermal sintering and ultra-fast pulsed light sintering. Due to the low decomposition temperature (~120 °C) of PVA substrates, the temperature during the thermal sintering process was selected to be 100 °C to avoid substrate damage. The highest conductivity of the resulting pattern is only 621.7 S/m after sintering on a hot plate for an extended period for over 3 h. The conductivity is only slightly higher than the as-printed sample, and is insufficient for practical applications. Photonic sintering of the patterns was conducted in a custom-made stainless-steel enclosure filled with argon using a photonic sintering system that generates a high intensity (4.9 J/cm^2^) wide spectrum light pulse (540 μs in duration). The photonic sintering process alone can lead to increased conductivity from 594.9 to 3237.5 S/m in one pulse without introducing the hot rolling process. When the hot rolling process is involved, the conductivity achieves 45,126.6 S/m after one pulse (Figure 2c) and a high conductivity of 60,213.6 S/m after twenty pulses. As the energy of the photonic sintering system is much lower than the one (25.88 J/cm^2^) in our previous publication [20], it is demonstrated that the effect of reduced sintering energy can be compensated by increasing the number of pulses (Figure 3(aIV)). Only slight changes in conductivity can be observed if the number of pulses keeps increasing. However, more pulses may lead to substrate damage, thus 20 pulses may be the optimum value for this particular sintering system. SEM images further confirm that no obvious change occur in the morphology of the patterns after over 20 pulses (Figure 3(aI–aII)), and the compactness of printed patterns is enhanced after multiple light pulses (Figure 2(aIV) and Figure 3(aI)). The results reveal that the surface of the printed pattern gradually becomes more compacted with increased number of pulses. It is also worth mentioning that dendrite formation is observed within the sintered sample due to rapid heating and cooling processes as analyzed in our previous publication [20]. However, the special flake shape of dendrite may indicate different surface energy and environmental conditions as compared with our previous work. The formation of these flakes may further reduce the distance between nanoparticles and increase the conductivity by offering increase contact areas.

The effect of surface oxidation is considered as one major obstacle for further improving the conductivity of bioresorbable patterns. Surface oxidation appears both as pre-existing surface layers that grow spontaneously during the synthesis of Zn NPs and as subsequent layers formed during the preparation and handling processes of pastes or inks. The surface composition has been studied using element EDS mapping after a photonic sintering process that were conducted under ambient environment without argon protection. It can be observed that oxygen has broad distribution in the tested sample and can correspond to 10 mol% Zn NPs (determined by EDS without any treatment, Figure 3b). The conductivity of such sample is only 39,581.3 S/m (Figure 2c) which is lower than that for the sample sintered in argon. Evidently, creating an oxygen-free environment is necessary in the photonic sintering process. In addition, previous studies have demonstrated a pre-existing thin layer of surface oxide on the as-synthesized Zn NPs [19,20,23]. This layer may be removed by acid treatment or reaction with reduction agents prior to use in an effort to obtain better conductivity.

**Figure 4:**
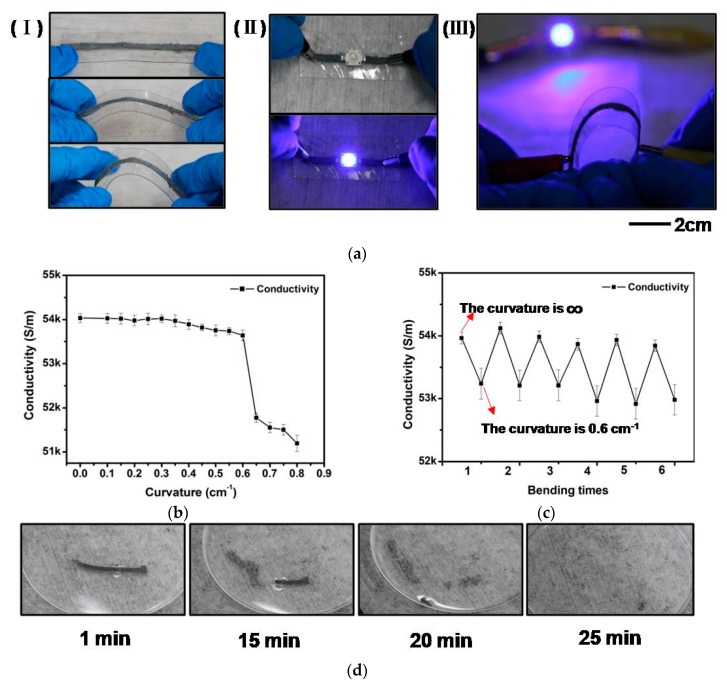
(**a**) (**I**) A printed pattern were bent into different curvatures; (**II**) The printed pattern as interconnects to connect a LED with a power supply; (**III**) An LED was lit by a printed interconnect in an extremely curved state; (**b**) The conductivity of a bioresorbable pattern in different curvatures; (**c**) Changes in conductivity when a bioresorbable pattern was bent repeatedly from 0 to 0.6 cm^−1^ in curvature; (**d**) A dissolution process of a printed pattern.

The flexibility and conductivity of the bioresorbable patterns were also explored. When bending a sintered pattern into different curvatures, no obvious damage and delamination can be observed (Figure 4(aI)). The conductivity of the pattern reduces continuously from 54,034.2 to 51,197.6 S/m with increased curvature from 0 to 0.8 cm^−1^. It is noticed that the conductivity is stable with less than 0.8% changes at curvatures ranging from 0 to 0.6 cm^−1^. Significant changes can be observed when the curvature becomes larger than 0.7 cm^−1^ (Figure 4b). This may be related to the formation of internal cracks due to applied bending strain. The reversibility of the printed patterns in response to repeated bending was conducted by bending the pattern from a planar state to a curved state with a curvature of 0.6 cm^−1^ repeatedly (Figure 4c). The results show reversible patterns in the conductivity with deviation between individual bending cycles for less than 1.5%. When connecting a light-emitting diode (LED) to the bioresorbable patterns (Figure 4(aII)), the LED can be lit after application of a power supply. The LED light was stable during the entire test process. When connecting the bioresorbable interconnect to a circuit that contained both a power supply and an LED, the LED could maintain its light intensity even when the interconnection was in an extremely curved state (Figure 4(aIII)), suggesting that the bioresorbable patterns may be able to adapt to complicate surrounding environments that demand large deformation.

Finally, the dissolution capability of the bioresorbable patterns was tested by immersing an unpackaged sample directly into DI water. The printed pattern started to dissolve rapidly after immersing in water. The entire dissolution process can be completed within 25 min (Figure 4d). The dissolution time can be fine-tuned by adding surface coating layers with various dissolution times.

## 4. Conclusions

This paper investigates several influential factors in determining the conductivity of bioresorbable patterns. Both an optimized composition and processing techniques have been achieved, leading to highly conductive bioresorbable patterns with a maximum conductivity of 60,213.6 S/m. The resulting patterns also possess excellent flexibility and dissolution capability. The experimental proof and approaches presented in this paper offer important guidelines for further improving the conductivity of bioresorbable pastes or inks that can adapt to the demands for mass fabrication and practical use in electronic industry.

Further study is required to minimize surface oxidation on nanoparticles. This may be tackled by introducing additives that can either absorb water to turn into weak acid that can gently remove surface oxidation layers on nanoparticles or work as reduction agents that may react with surface oxidation when exposed to pulsed sintering light. In addition, complex circuit traces with more electronic components may be achieved using screen printing and ink dispersion, resulting in integrated bioresorbable PCB circuits that may be able to replace current build-to-last circuits in many applications.
